# Plasmon-Enhanced Light Absorption in GaAs Nanowire Array Solar Cells

**DOI:** 10.1186/s11671-015-1110-1

**Published:** 2015-11-06

**Authors:** Yanhong Li, Xin Yan, Yao Wu, Xia Zhang, Xiaomin Ren

**Affiliations:** State Key Laboratory of Information Photonics and Optical Communications, Beijing University of Posts and Telecommunications, Beijing, 100876 China

**Keywords:** Solar cells, Surface plasmon, Nanowire arrays, Nanoparticle, Semiconductors

## Abstract

In this paper, we propose a plasmon-enhanced solar cell structure based on a GaAs nanowire array decorated with metal nanoparticles. The results show that by engineering the metallic nanoparticles, localized surface plasmon could be excited, which can concentrate the incident light and propagate the energy to nanowires. The surface plasmon can dramatically enhance the absorbance of near-bandgap light, and the enhancement is influenced by the size and material of nanoparticles. By optimizing the particle parameters, a large absorbance enhancement of 50 % at 760 nm and a high conversion efficiency of 14.5 % can be obtained at a low diameter and period ratio (*D*/*P* ratio) of 0.3. The structure is promising for low-cost high-performance nanoscale solar cells.

## Background

The development of high-efficiency photovoltaic (PV) systems has always been a topic of intensive research to solve future energy problems. However, to make power from photovoltaics competitive with fossil fuel technologies and for widespread implementation, the cost needs to be reduced by a factor of 2–5 [1]. Many approaches have been proposed to reduce the cost of photovoltaics, such as nanowire array (NWA) devices replacing film ones, or use metallic nanostructures that support surface plasmon to improve light absorption.

Semiconductor NWAs are presently under intensive research and development for next-generation solar cells due to their low cost and high conversion efficiency compared with conventional thin-film devices [2–5]. GaAs nanowires show particular promise due to the superior electrical and optical properties, such as direct bandgap and high absorption coefficient. On the other hand, plasmonic nanostructures are believed to enhance light trapping and have recently been used in photodetectors [6, 7], photodiodes [8, 9], and solar cells [10–17] including Si solar cells [10–14] and organic cells [15, 16]. However, the combination of plasmon within III-V solar cells has been rarely reported, much less in III-V NWAs. In this work, we introduce plasmon into GaAs NWA solar cells to improve the photoconversion efficiency at a low *D*/*P* ratio. It has been proved that the NWA geometry can dramatically enhance the light absorption [18]. However, we have found in this paper that as the *D*/*P* decreases, the trapping ability of NWA attenuates, especially for near-bandgap light, and by engineering the metallic nanoparticles, localized surface plasmon would be excited, which can concentrate light around and propagate energy to GaAs NWs to improve the absorbance of near-bandgap light. Thus, a relatively high absorptance of light can be obtained at a low *D*/*P* ratio, dramatically reducing the material cost for the device. Finite-difference time-domain (FDTD) simulation is used to study the influence of plasmon on the light trapping and optical absorption of GaAs NWAs, while the optical generation and photoconversion efficiency are evaluated through Sentaurus Electromagnetic Wave (EMW) Solver module package.

## Methods

**Fig. 1:**
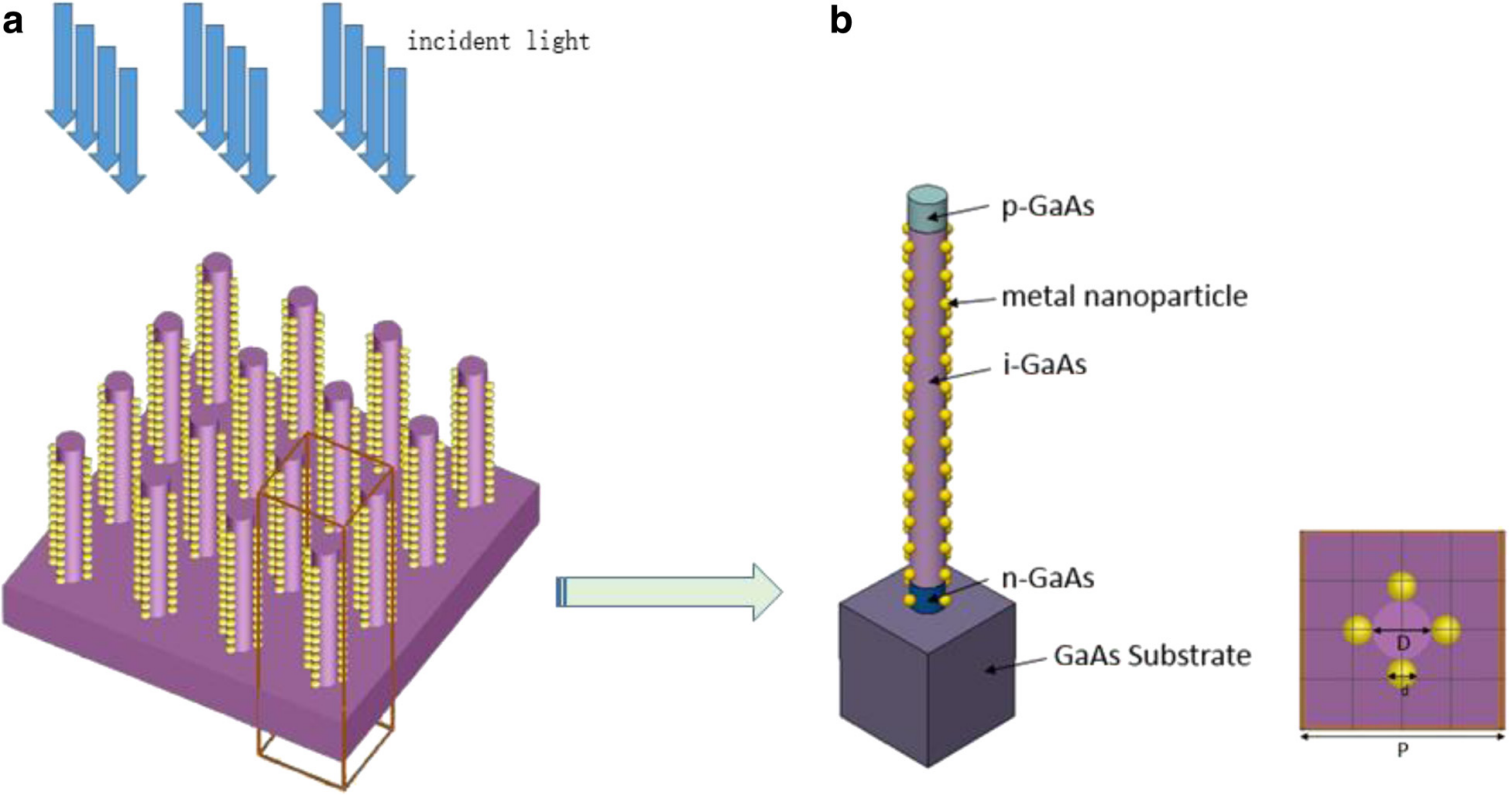
3-D illustration and schematic drawing of the proposed structure. **a** Schematic drawing of vertically aligned NW arrays decorated with metal nanoparticles. **b** 3-D illustration of the proposed NW structure decorated with nanoparticles in axially p-n junction NW

The proposed structure is illustrated in Fig. 1. The structure consists of a GaAs NWA with diameter *D* and period *P* decorated by gold nanoparticles with diameter *d* on the sidewall arranged in a square lattice and surrounded by air. No metal nanoparticles are placed in the p-GaAs region, as the metal nanoparticles on the top of NW would catch most incident light, but the carrier generated in the p-GaAs region cannot be collected by electrode effectively. The wavelength-dependent complex refractive index used to describe the material dispersion properties of GaAs can be obtained from the study of Levinshtein et al. [19]. By applying periodic boundary conditions in the *x* and *y* directions, the simulations are carried out within this unit cell to model the periodic NWA structure. The simulation domain is closed at the top and bottom with a perfectly matched layer, allowing reflected light and transmission light to escape the simulation volume. The incident light from the top is set in parallel to the NW axis as indicated in Fig. 1a, and we use a plane wave defined with power intensity and wavelength values from a discretized AM 1.5G solar spectrum with a wavelength ranging from 290 to 900 nm (typical absorption region of GaAs) to model the sunlight. The reflection monitor is located at above the top surface of the NWA, and the transmission monitor is located at the bottom surface of substrate to calculate the light absorbed. The amount of power transmitted through the power monitors is normalized to the source power at each wavelength. The reflectance *R*(*λ*) and transmission *T*(*λ*) are calculated by the equation

$$ R\left(\lambda \right),\;T\left(\lambda \right)=0.5\;{\displaystyle \int \mathrm{real}\left\{p{\left(\lambda \right)}_{\mathrm{monitor}}\right\}}dS/{P}_{\mathrm{in}}\left(\lambda \right) $$

where, *p*(*λ*) is the Poynting vector, *dS* is the surface normal, and ${P}_{\mathrm{in}}\left(\lambda \right)$ is the incident source power at each wavelength. The absorption spectrum *A*(*λ*) of the GaAs NWAs is given by the equation

$$ A\left(\lambda \right)=1-R\left(\lambda \right)-T\left(\lambda \right) $$

For the electrical modeling partial simulated by Sentaurus Electromagnetic Wave (EMW) Solver and Sdevice Solver module package, the 3-D optical generation profiles are incorporated into the finite-element mesh of the NWs in the electrical tool [20, 21]. The device electrical simulation takes the doping-dependent mobility (GaAs only) and bandgap narrowing and radiative, Auger, and Shockley-Reed-Hall (SRH) recombinations into consideration. The material parameters critical for device simulations are mostly obtained from the Levinshtein model, which is shown in Table 1. The Arora model [21, 22] is adopted in the calculation of the doping-dependent mobility, which reads

**Table 1:** Key material parameters

Parameters	Values (GaAs)
Minimum mobility (cm^2^/V · s)	2.136 × 10^3^ (21.48)
SRH lifetime (ns)	1 (1)
Effective density of states (/cm^3^)	4.42 × 10^17^ (8.47 × 10^17^)
Auger coefficient (cm^6^/s)	1.9 × 10^−31^ (1.2 × 10^−31^)
Surface recombination velocity (cm/s)	10^7^ (10^7^)

Unless mentioned specifically, all simulations in this work use the parameters in this table by default. The numbers in the front and in the parentheses are for the electrons and holes, respectively

$$ {\upmu}_{\mathrm{dop}}={\mu}_{\min }+\frac{\mu_d}{1+{\left(N/{N}_0\right)}^A} $$

where *A* is 0.6273 (0.8075) and *N*_0_ is 7.345 × 10^16^ (5.136 × 10^16^)/cm^3^ for the electrons (holes). The current-voltage relationship is calculated by

$$ J={J}_{\mathrm{SC}}-{J}_0\;\left({ \exp}^{V/V\mathrm{c}}-1\right) $$

where $J$ is the current density of the solar cell, ${J}_{\mathrm{sc}}$ is the photocurrent density, ${J}_0$ is the reverse saturation current density, $V$ is the voltage between the terminals of the cell, and ${V}_{\mathrm{c}}$ is the thermal voltage, which can be given by

$$ {V}_{\mathrm{c}}=\frac{K_{\mathrm{B}}{T}_{\mathrm{c}}}{q} $$

in which ${K}_{\mathrm{B}}$ is the Boltzmann constant, *T*_c_ is the cell temperature, and *q* is the elementary charge.

There are two ways to import metal particles in experiment: we cover the nanowire array by a metal film with specific thickness, and approximate hemisphere metal particles can be obtained by thermal annealing treatment. The particles’ size can be determined by the thickness of the metal film. We can also import golden particles by spin coating gold nanosol over nanowire arrays. In this method, confecting specific gold nanosol to meet the experimental requirements is the key point and the metal particles we get are in spherical shape. In this paper, all of the metal particles have spherical shapes referring to the latter method.

## Results and Discussion

**Fig. 2:**
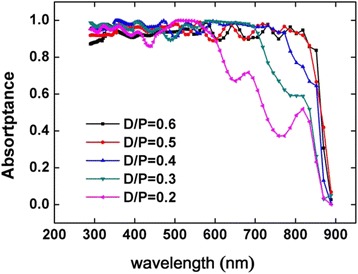
The absorption of NWAs with different *D*/*P* without metal nanoparticles

Figure 2 displays the absorptance spectra at different *D*/*P* ratios. We find that the best nanowire diameter is different at different *D*/*P* ratios, and we optimize the diameter for matching the *D*/*P* ratio to gain the largest absorptance. It has been demonstrated that the *D*/*P* ratio plays an important role in NWA absorption, and there is an optimized structure parameter [18]. From Fig. 2, we can see that all simulations have a nice absorption under 550 nm, and the *D*/*P* ratio has little effect in the short wavelength range. However, the *D*/*P* ratio has a great influence on the absorptance at near-bandgap wavelength. We get a perfect absorption spectrum at a *D*/*P* ratio of 0.5, and the absorptance changes little when we continue to increase the *D*/*P* ratio. But, when we reduce the *D*/*P*, the near-bandgap absorptance drops obviously. The results indicate that the light trapping effect of NWAs dramatically decreases at a low filling ratio at near-bandgap wavelength.

**Fig. 3:**
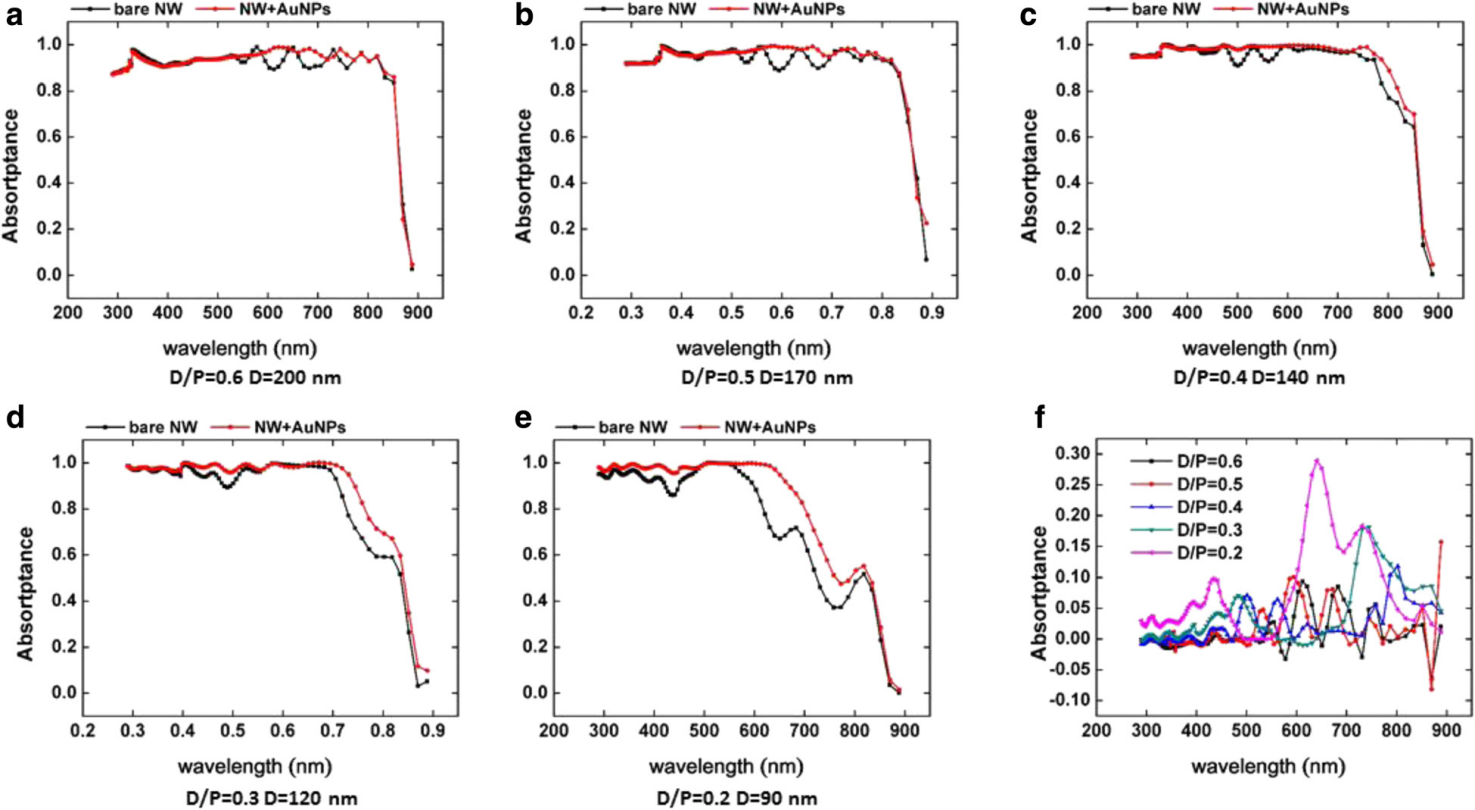
Influence of gold nanoparticles to different *D*/*P* ratios of NWAs on light absorption. The nanoparticle diameter is 40 nm, and the period is 100 nm

Figure 3 shows the absorptance spectra of NWAs decorated with Au nanoparticles compared to bare ones. It can be seen from the figure that the nanoparticles have little influence to the absorptance of the NWAs with high nanowire density, when the *D*/*P* is 0.6 and 0.5. But, when we reduce the density of nanowires, the absorptance of NWAs at near-bandgap wavelength improved by metal nanoparticles more and more obviously. Comparing pictures c, d, and e in Fig. 3, we can conclude that the metal particle does improve the absorptance of GaAs NWAs when the *D*/*P* ratio is small, and the improvement gets higher when the *D*/*P* ratio reduces. The last picture f in Fig. 3 shows the enhanced absorption at different *D*/*P* ratios, calculated by the absorption of NWAs decorated with Au NPs minus the absorption of bare NWAs.

**Fig. 4:**
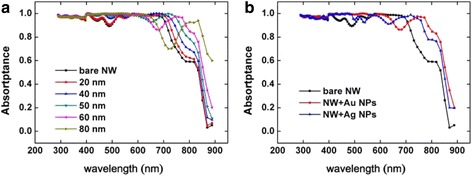
The absorptance with different metal nanoparticles. **a** The absorptance of NWAs (*D* = 120 nm, *P* = 400 nm, *D*/*P* = 0.3) with different size (period is 100 nm, diameter *d* from 20 to 80 nm) gold nanoparticles. **b** The absorptance of NWAs (*D* = 120 nm, *P* = 400 nm, *D*/*P* = 0.3) with different material (Au, Ag) nanoparticles (period is 100 nm, diameter is 60 nm)

The size and material of the metal nanoparticles can strongly influence the coupling efficiency of surface plasmon [1]. Figure 4 shows the absorptance of GaAs NWs decorated with metal nanoparticles with varying sizes and different metals. We select silver nanoparticles compared with gold nanoparticles, which have widely been considered for plasmonic photovoltaics. The *D*/*P* ratio is set to be 0.3, and the diameter of NWs is set to be 120 nm. The diameter of Au NPs is set to be 20, 40, 50, 60, and 80 nm, respectively. From Fig. 4a, we can see that the absorptance is enhanced as the Au size increases at the near-bandgap wavelength around 800 nm. We can see that the absorptance spectrum reduces around 700 nm, and following NP diameter increase, the absorptance gets lower around 700 nm. The reflection monitor gets a high reflection at this band. This is because of the light scattering effect of metal nanoparticles. So, the size of the nanoparticle is not the larger the better.

Next, we set the diameter of nanoparticles at 60 nm, the *D*/*P* of NWAs at 0.3, and the diameter of NWs at 120 nm and study the influence of the material of nanoparticles on the absorptance is displayed in Fig. 4b. We can see that NWAs decorated with the two types of nanoparticles both have an obvious absorption enhancement at a wavelength from 700 to 900 nm. The absorptance spectra below 500 nm are similar for the two different metals, but the scattering wavelength range is different. The reflection spectrum with Au nanoparticles is larger than Ag, but the absorptance enhancement around 800 nm is larger at the same time. The Au nanoparticles and the Ag nanoparticles have the scattering peak which led to higher reflection and lower absorption of incident light.

**Fig. 5:**
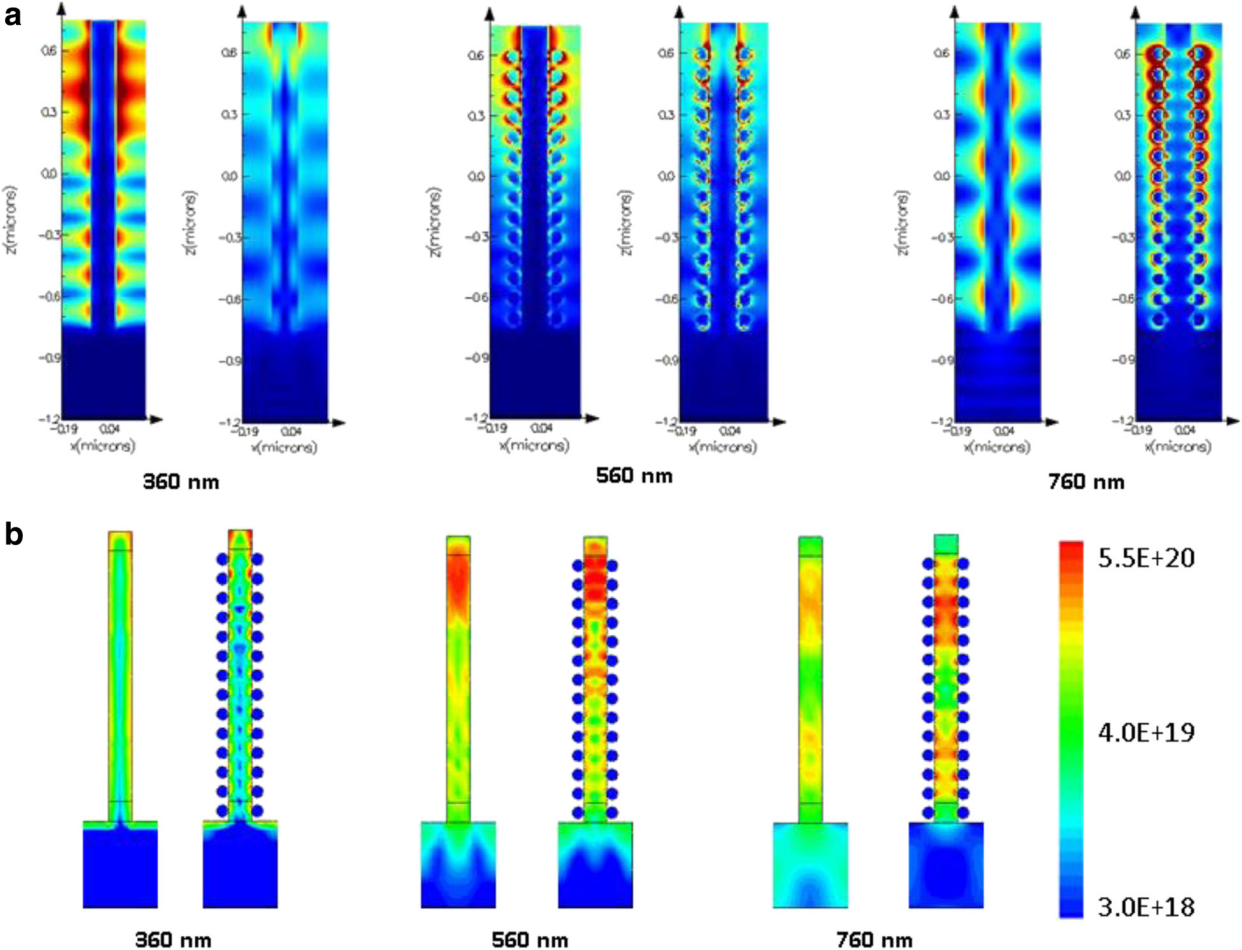
The light distribution and optical generation in nanowire. **a** Light distribution in NWAs at wavelengths 360, 560, and 760 nm. **b** The optical generation distribution density of NWA structure with Au NPs in contrast to bare ones at 360, 560, and 760 nm

To clarify the light distribution affected by the surface plasmon, we choose three typical wavelengths and compare the light distribution inside the structure, shown in Fig. 5a. We can see that, at short wavelengths like 360 and 560 nm, GaAs NWA has a nice absorptive character, and the incident light is mostly absorbed at the top of NWAs. The localized surface plasmon resonances (LSPRs) excited in a near-infrared spectrum have a rather smaller extinction coefficient [23], and the nanoparticles do not have much improvement to light absorption. On the contrary, the absorptance at 760 nm is improved obviously with metal nanoparticles. Light field of bare NWAs is almost unchanged from the top to the substrate, which means that little light is absorbed in bare NWAs. When ( LSPRs) are introduced, the light field around Au nanoparticles is strong. Light can be coupled from the Au NPs into neighboring GaAs NWs, which leads to a remarkable portion (above 90 %) of incident light absorbed, much higher than that in conventional structure (less than 60 %). In this situation, the nanoparticles act as an effective “antenna” for the incident sunlight, which stores the incident energy in a localized surface plasmon mode.

Next, we import our structure and parameters into Sentaurus Electromagnetic Wave (EMW) Solver and Sdevice module package to investigate the optical generation and photoconversion efficiency. Figure 5b displays the optical generation at 360, 560, and 760 nm. At 360 nm, the photocarrier is mainly generated at the surface of NWs, showing a fairly short absorption length due to the high absorption capacity of GaAs. The gold nanoparticles increase the absorption at the surface layer and lead to lower photocarrier generation in inner NWs. At a slightly longer wavelength of 560 nm, the majority of light is absorbed at the top of NWs, and the gold nanoparticles not only increase the photocarrier generation at the top but also gather light at the bottom and increase photocarrier density. At 760 nm, as the absorption coefficient of GaAs is only 0.096, the density of the photocarrier is quite low in bare NWAs, but the density of the photocarrier of NWAs with gold nanoparticles is much higher as the LSPR effect is excited by metal nanoparticles.

**Fig. 6:**
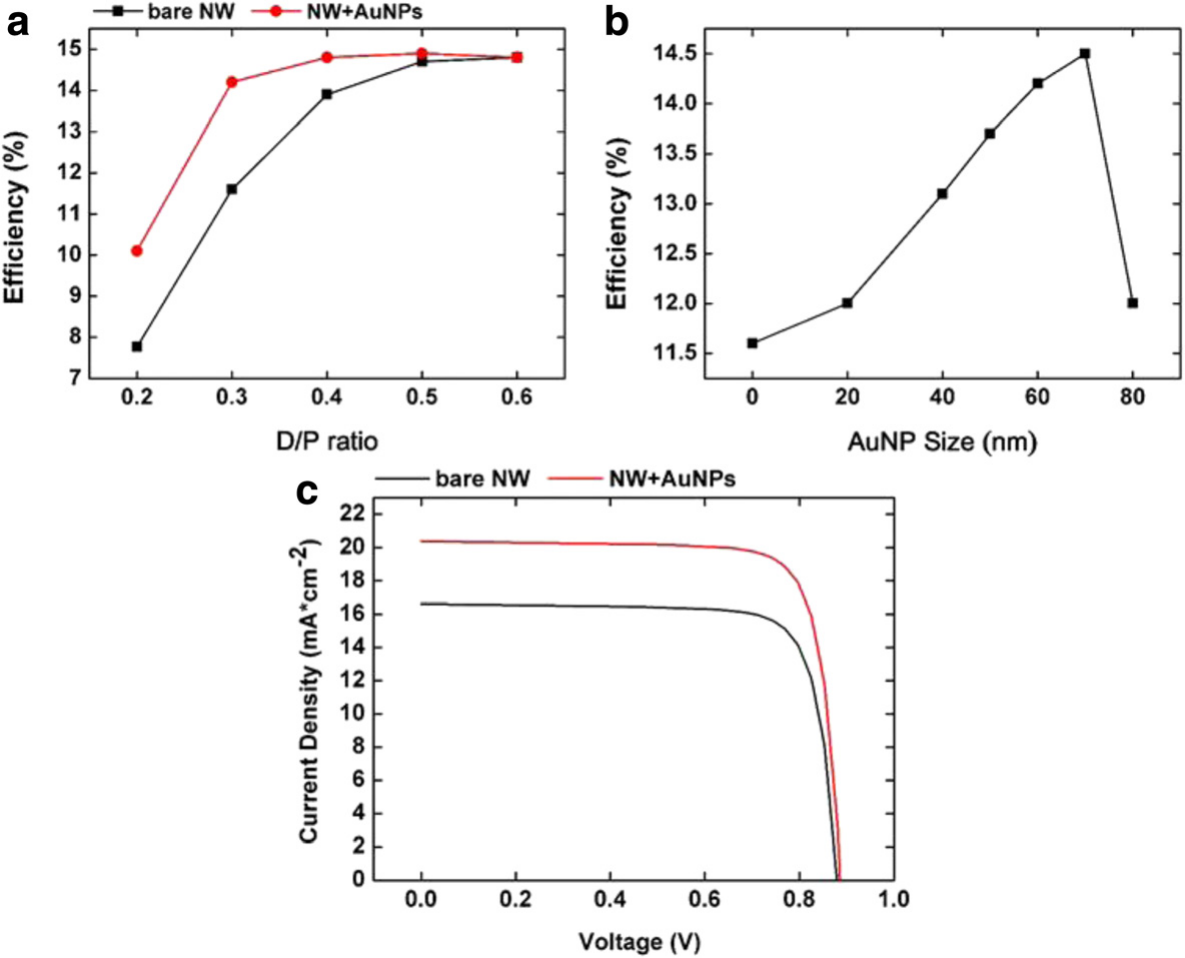
The photoconversion efficiency and *I*-*V* curve. **a** The photoconversion efficiency of NWAs with different *D*/*P* decorated by 60-nm Au nanoparticles. **b** The photoconversion efficiency of NWAs with *D*/*P* = 0.3 and Au nanoparticle size from 0 to 80 nm. **c** Comparison of *I*-*V* characteristics between bare NWAs and NWAs with 35-nm Au NPs

Figure 6a shows the efficiency of NWA solar cell under AM 1.5G illuminations. We can see that the photoconversion efficiency increases with the increase of *D*/*P* ratio when *D*/*P* ratio is under 0.5. If the *D*/*P* ratio is larger than 0.5, the photoconversion efficiency does not continue to increase. This is because that, when the *D*/*P* ratio is large enough, the incident light could be totally absorbed at full wavelength band by GaAs nanowires. On the other hand, the reflection becomes larger at high *D*/*P* ratios, which would reduce the efficiency of absorption. We can see from the figure that when the *D*/*P* is 0.3, the efficient of new structure is improved from 11.6 to 14.2 % by 22 %. Figure 6b shows the photoconversion efficiency with different sizes of gold particles. We can see that the efficiency is higher for larger nanoparticles smaller than 70 nm. When the gold nanoparticle further increases to 80 nm, the efficiency drops as the scattering effect increases the reflection of NWAs and reduces the absorptance spectrum just like Fig. 4 shows.

**Fig. 7:**
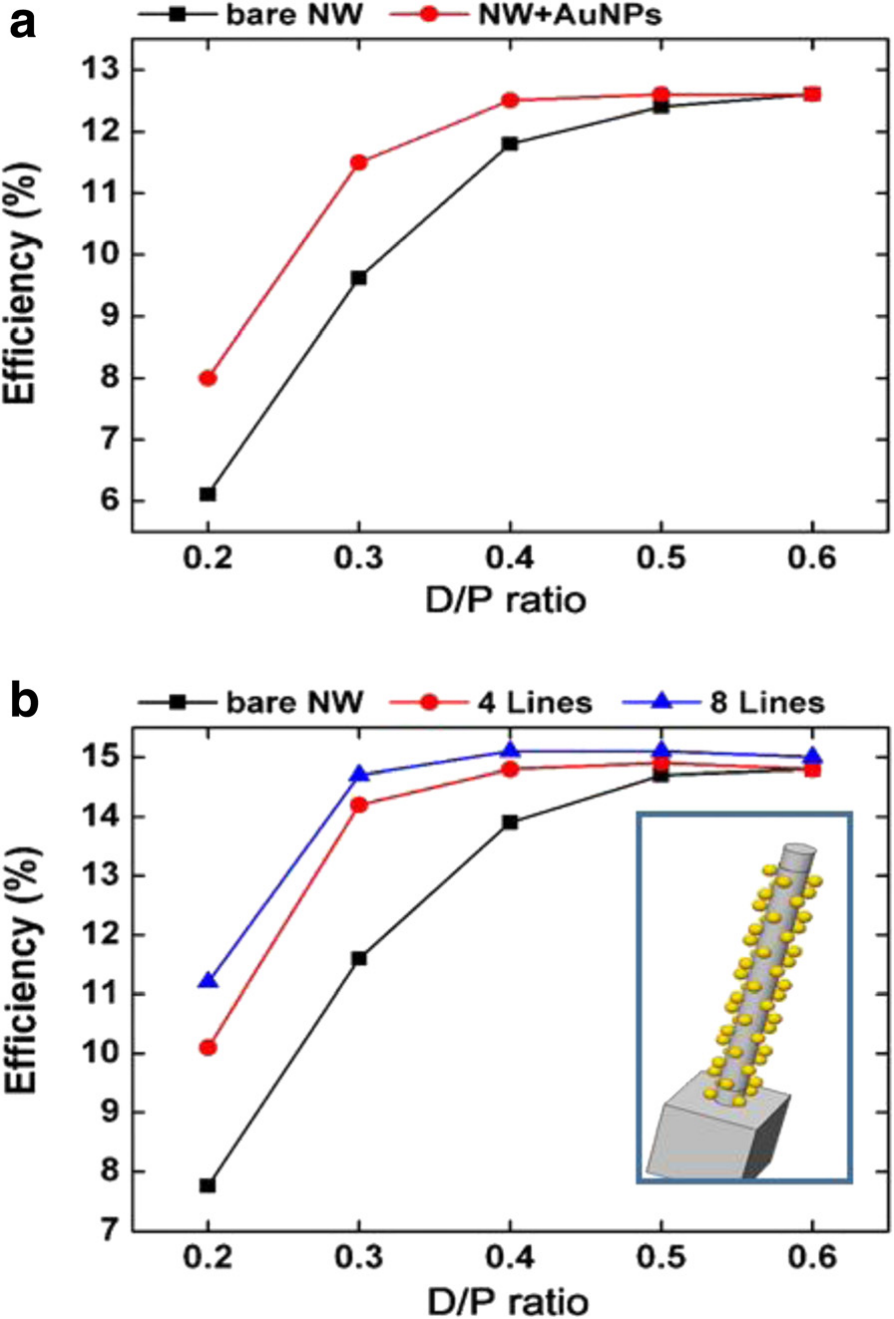
The photoconversion efficiency of oblique incidence and equidistribution. **a** The photoconversion efficiency of NWAs with different *D*/*P* decorated by 60-nm Au nanoparticles with 30° angle of incidence. **b** Comparison of the efficiency of nanowire arrays with difference particle layouts. *Inset* shows the nanowire model with metal particles arranged in eight lines

As the incident angle has a great influence on photovoltaic performance, oblique incidence will increase scattering cross section which will affect the photoelectric conversion efficiency observably. So, we adjust the angle of incidence to 30° off perpendicular direction, and the result is shown in Fig. 7a. When we compare the result with Fig. 6a, we can find that the efficiency decreases in all of the considered structures when oblique incidence. But We can see from the picture that the photoconversion efficiency of nanowire arrays with metal particles isstill better than their bare counterparts obviously.

In experiment, it is tough to arrange the metal particles in four lines strictly with same separation distance periodically and it is tough to get a random distribution in simulation But, the equidistribution would be the perfect state. So we continue our simulation by change the metal particles distribution mode as Fig. 7b. The number of metal particles is unaltered, while they are arranged in eight lines with their interval into double. The efficiency of solar cells is shown in Fig. 7c. Simulation result shows the photoelectric conversion efficiency is higher with equidistribution. So, in experiment, we should find effective methods to make sure metal particles distribution uniformly.

The current density versus voltage (*I*-*V*) characteristics of NWA solar cells are shown in Fig. 6c. Compared with the NWA solar cell without gold nanoparticles, the open circuit voltage (*V*_oc_) with gold nanoparticles slightly increases from 0.879 to 0.886 V, while the short circuit current density (*J*_sc_) dramatically increases from 16.63 to 20.38 mA/cm^2^. The enhancement of photocurrent density with gold nanoparticles in our experiment was obvious, and we just provide a method to improve the light trapping in lower *D*/*P* ratios to save the GaAs material. Our study which combines the LSPRs with nanowire arrays, both have evident effect in trapping light, provides some foreshadowing to further research to reduce the cost of solar cells.

## Conclusions

In summary, we proposed a novel plasmon-enhanced solar cell structure based on a GaAs nanowire array decorated with metal nanoparticles. This structure combines LSPRs with nanowire arrays together to reduce the cost of solar cells. A 3-D employed FDTD method in optical simulation and optoelectronic simulation is used to evaluate the performance of the metal nanoparticles decorated on the sidewalls of NWs in GaAs NWAs. The results show that by engineering the metallic nanoparticles, localized surface plasmon could be excited, which can concentrate the incident light and propagate the energy to nanowires. By optimizing the particle parameters, a large absorbance enhancement of 50 % at 760 nm and a high conversion efficiency of 14.5 % can be obtained at a low *D*/*P* ratio of 0.3. The structure is promising for low-cost high-performance nanoscale solar cells.

## References

[1] Atwater HA, Polman A (2010). Plasmonics for improved photovoltaic devices. Nat Mater.

[2] Wu J, Yu P, Susha AS, Sablon KA (2015) Broadband efficiency enhancement in quantum dot solar cells coupled with multispiked plasmonic nanostars. Nano Energy 13:827–835.

[3] Zhu J, Yu Z, Burkhard GF, Hsu C-M, Connor ST, Xu Y, Wang Q, McGehee M, Fan S, Cui Y (2009). Optical absorption enhancement in amorphous silicon nanowire and nanocone arrays. Nano Lett.

[4] Hu L, Chen G (2007). Analysis of optical absorption in silicon nanowire arrays for photovoltaic applications. Nano Lett.

[5] Garnett E, Yang PD (2010). Light trapping in silicon nanowire solar cells. Nano Lett.

[6] Stuart HR, Hall DG (1996). Absorption enhancement in silicon-on-insulator waveguides using metal island films. Appl Phys Lett.

[7] Stuart HR, Hall DG (1998). Island size effects in nanoparticle-enhanced photodetectors. Appl Phys Lett.

[8] Lim SH, Mar W, Matheu P, Derkacs D, Yu ET (2007). Photocurrent spectroscopy of optical absorption enhancement in silicon photodiodes via scattering from surface plasmon polaritons in gold nanoparticles. J Appl Phys.

[9] Schaadt DM, Feng B, Yu ET (2005). Enhanced semiconductor optical absorption via surface plasmon excitation in metal nanoparticles. Appl Phys Lett.

[10] Beck FJ, Polman A, Catchpole KR (2009). Tunable light trapping for solar cells using localized surface plasmons. J Appl Phys.

[11] Pillai S, Catchpole KR, Trupke T, Green MA (2007). Surface plasmon enhanced silicon solar cells. J Appl Phys.

[12] Temple TL, Mahanama GDK, Reehal HS, Bagnall DM (2009). Influence of localized surface plasmon excitation in silver nanoparticles on the performance of silicon solar cells. Sol Energy Mater Sol Cells.

[13] Derkacs D, Lim SH, Matheu P, Mar W, Yu ET (2006). Improved performance of amorphous silicon solar cells via scattering from surface plasmon polaritons in nearby metallic nanoparticles. Appl Phys Lett.

[14] Losurdo M, Giangregorio MM, Bianco GV, Sacchetti A, Capezzuto P, Bruno G (2009). Enhanced absorption in Au nanoparticles/a-Si:H/c-Si heterojunction solar cells exploiting Au surface plasmon resonance. Sol Energy Mater Sol Cells.

[15] Jeong JA, Kim HK (2009). Low resistance and highly transparent ITO–Ag–ITO multilayer electrode using surface plasmon resonance of Ag layer for bulk-heterojunction organic solar cells. Sol Energy Mater Sol Cells.

[16] Westphalen M, Kreibig U, Rostalski J, Luth H, Meissner D (2000). Metal cluster enhanced organic solar cells. Sol Energy Mater Sol Cells.

[17] Min CJ, Li J, Veronis G, Lee JY, Fan SH, Peumans P (2010). Enhancement of optical absorption in thin-film organic solar cells through the excitation of plasmonic modes in metallic gratings. Appl Phys Lett.

[18] Haomin G, Long W, Xinhua L, Zhifei Z, Yuqi W (2011). Analysis of optical absorption in GaAs nanowire arrays. Nanoscale Res Lett.

[19] Levinshtein M, Rumyantsev S, Shur M (1999). Handbook series on semiconductor parameters, ternary and quaternary III-V compounds.

[20] Sijia W, Xin Y, Xia Z, Junshuai L, Xiaomin R (2015). Axially connected nanowire core-shell p-n junctions: a composite structure for high-efficiency solar cell. Nanoscale Res Lett.

[21] Synopsys (2013). Sentaurus device user guide (version G-2013.03).

[22] Arora ND, Hauser JR, Roulston DJ (1982). Electron and hole mobilities in silicon as a function of concentration and temperature. Electron Devices, IEEE Trans.

[23] Catchpole KR, Polman A (2008). Plasmonic solar cells. Opt Express.

